# Transcatheter arterial chemoembolization is safe and effective for patients with late-stage or recurrent oral carcinoma

**DOI:** 10.3389/fonc.2022.831583

**Published:** 2022-07-22

**Authors:** Yonghua Bi, Tianfeng Du, Wenting Pan, Fan Tang, Yang Wang, Dechao Jiao, Xinwei Han, Jianzhuang Ren

**Affiliations:** ^1^ Department of Interventional Radiology, The First Affiliated Hospital of Zhengzhou University, Zhengzhou, China; ^2^ Department of Stomatology, The First Affiliated Hospital of Zhengzhou University, Zhengzhou, China

**Keywords:** TACE, oral carcinoma, oral hemorrhage, complications, oxaliplatin, raltitrexed

## Abstract

**Objective:**

We reported the long-term outcomes of transcatheter chemoembolization (TACE) for patients with late-stage or recurrent oral carcinoma.

**Methods:**

This retrospective study enrolled 18 patients with late-stage or recurrent oral carcinoma between December 2015 and April 2021. The tumor-feeding artery was catheterized, and cisplatin/oxaliplatin and 5-FU/raltitrexed were infused with embolization using polyvinyl alcohol or gelatin sponge. Computed tomography was performed at about 1, 3, and 6 months after the procedure, and every 6 months after that. During the procedure and follow-up, procedure outcomes, complications, treatment efficacy, and overall survival were analyzed.

**Results:**

A total of 31 sessions of TACE were performed, with a technical success rate of 100%. Of 12 patients combined with oral hemorrhage, two patients showed rebleeding 35 and 37 days later, with a clinical efficiency of hemostasis of 88.9%. Mild complications were observed in 11 patients (61.1%). Severe complications or procedure-related deaths were not observed during or after the procedure. The objective response rate and disease control rate were 20.0% and 86.7%, 38.5% and 61.5%, and 25.0% and 50.0% at 1, 3, and 6 months later, respectively. Seventeen patients (94.4%) were followed up, with a median duration of 37.8 months (IQR 22.3–56.8). Nine patients died of tumor progression, one died of massive rebleeding, and one died of severe lung infection. The median overall survival was 23.8 months.

**Conclusion:**

TACE is a safe and effective procedure with minimal invasiveness for treating late-stage or recurrent oral carcinoma. TACE can be recommended as a palliative treatment, particularly for patients with oral hemorrhage.

## Introduction

As one of the most common carcinomas in the head and neck, about half of the patients with oral carcinoma are diagnosed or treated at their late stage, resulting in a poor prognosis. Oral carcinoma can be effectively managed by traditional surgery. However, surgical resection may affect facial appearance and damage oral function, and tumor recurrence may be inevitable for some patients. For late-stage inoperable patients, conventional chemoradiotherapy can be used as a palliative treatment and targeted therapy has been carried out as a novel management (1). Unfortunately, those patients often suffer side effects and adverse events after receiving palliative treatment.

Transcatheter chemoembolization (TACE) has been widely performed for the palliative treatment of late-stage carcinomas (2, 3), including advanced head and neck cancers (4–6). Besides, preoperative TACE has also been used to decrease tumor size to improve surgical success rate or reduce the recurrence rate of postoperative tumors (7, 8). To date, few studies that have reported the clinical outcomes of TACE for treating late-stage or recurrent oral carcinoma (6, 9). In this study, we reported the long-term outcomes of TACE for treating patients with late-stage or recurrent oral carcinoma.

## Materials and methods

### Patients

**Figure 1:**
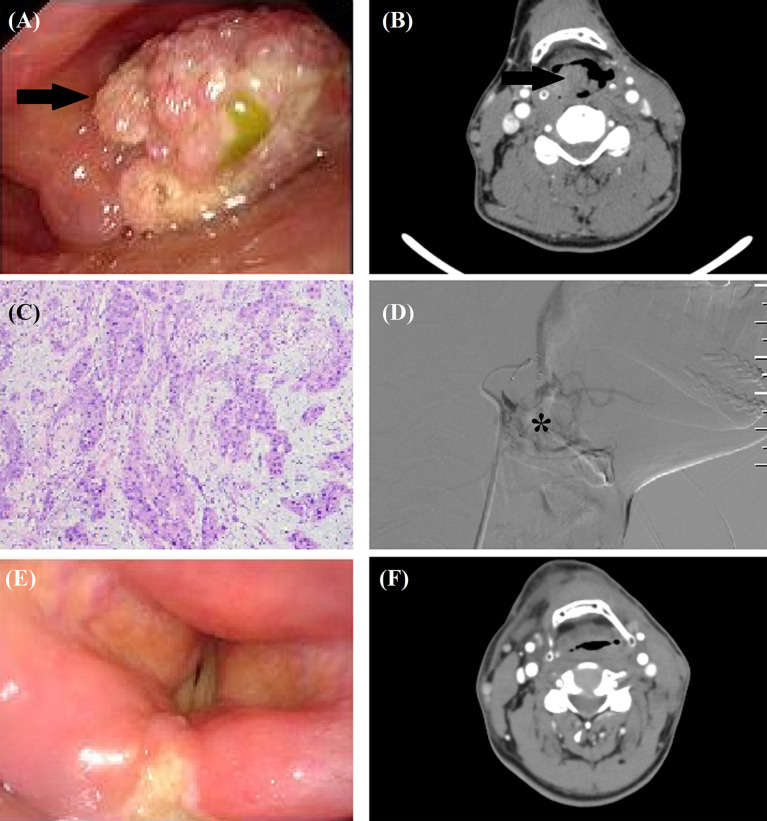
TACE for a 56-year male with recurrent squamous cells carcinoma after tongue cancer resection. **(A, B)** A recurrent tumor (arrow) was shown by laryngoscopy and computed tomography. **(C)** The tumor was histopathologically diagnosed as squamous cells carcinoma. **(D)** Tumor staining (*) was shown by angiography. **(E, F)** Laryngoscopy and computed tomography confirmed that the tumor disappeared 7 months after TACE.

**Figure 2:**
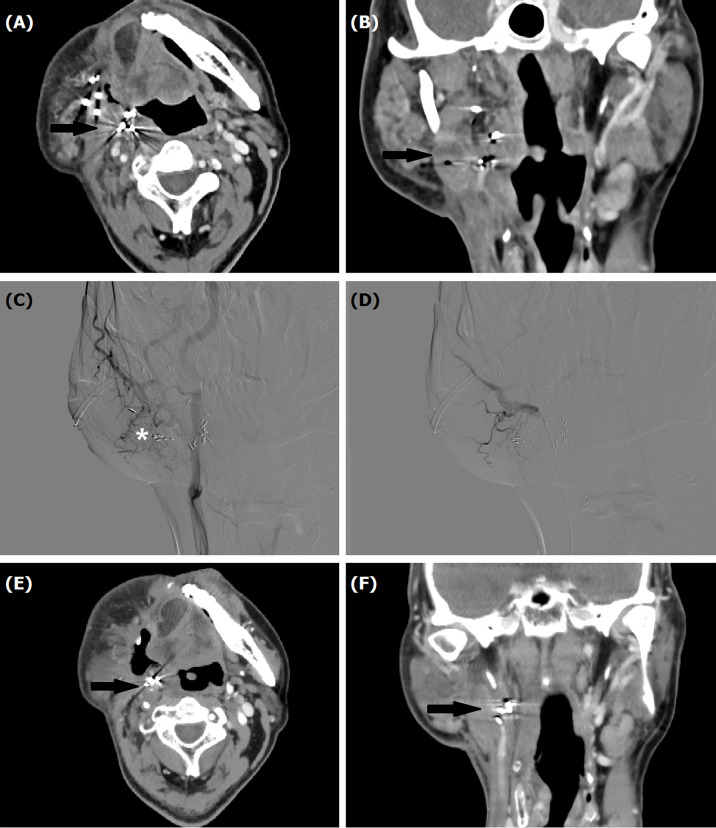
A 53-year male treated by TACE for recurrent mucoepidermoid carcinomas in tongue. **(A, B)** Computed tomography showed a recurrent tumor with Iodine-125 seeds (arrow). **(C, D)** Tumor staining (*) was shown by angiography, which disappeared after TACE. **(E, F)** A decreased tumor (arrow) was shown by computed tomography examination about 1 month later.

**Figure 3:**
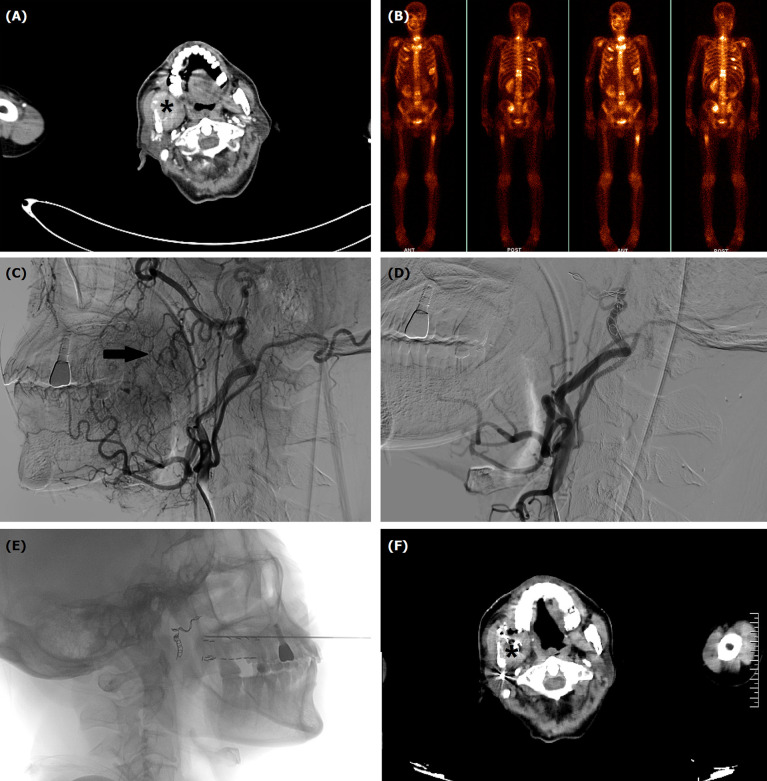
TACE for a 63-year male with oral hemorrhage due to recurrent squamous cells carcinoma in mandible and maxilla. **(A)** Computed tomography showed a tumor (*) in right mandible and maxilla. **(B)** Numerous bone metastases were visible in systemic bone imaging. **(C)** Tumor staining (arrow) and varies blood vessels were shown by angiography. **(D)** The tumor staining and tumor-feeding arteries disappeared after TACE. **(E)** Iodine-125 seeds implantation was performed about 1 month after TACE. **(F)** The tumor (*) decreased 4 months after TACE.

This retrospective study included 18 patients with oral carcinoma between December 2015 and April 2021. Including criteria were 1) histopathologically diagnosed as oral carcinoma, both primary and recurrent carcinoma (**Figures 1A–C**); 2) being of stages IIIA–IV (**Figures 2A, B**, **3A, B**); 3) estimated survival >3 months; 4) without severe dysfunction in the heart, liver, and kidney; and 5) white blood cells >3 × 10^9^/L. There were 11 male and seven female patients, including tongue cancer (n = 5), gingival cancer (n = 2), carcinoma of the mouth floor (n = 6), palate carcinoma (n = 2), and cancer of the mandible/maxilla (n = 3). The ages of the patients ranged from 26 to 84 years, with a median age of 58.5 years. There were 10 recurrent cases after surgical resection, and the remaining eight cases had an initial onset. Only five patients had no metastases. The remaining patients showed lymphatic (n = 8), liver (n = 3), bone (n = 3), and lung metastases (n = 2), respectively. Twelve patients showed massive oral bleeding on admission (**Table 1**). Ethical approval was waived by the Institutional Review Board of the University due to its retrospective nature. Written informed consent was obtained from all patients before the TACE procedure.

**Table 1:** Baseline clinicopathological characteristics of the study patients.

Variables	TACE
Number of patients	18
Mean age, years	58.5 (46.0, 68.8)
Gender, male	11 (61.1%)
Disease course, months	11.0 (4.5, 24.0)
Pathological types of tumors	
Squamous cells carcinoma	13 (72.2%)
Mucoepidermoid carcinomas	1 (5.6%)
Adenocarcinoma	1 (5.6%)
Others	3 (16.7%)
The sites of tumors	
Tongue	5 (27.8%)
Gingival	2 (11.1%)
Floor of mouth	6 (33.3%)
Palate	2 (11.1%)
Mandible/maxilla	3 (16.7%)
Recurrence after surgery	10 (55.6%)
Radiochemotherapy	14 (77.8%)
Oral hemorrhage	12 (66.7%)

### TACE Procedure

All procedures were performed under local anesthesia and fluoroscopical guidance. The femoral artery was punctured using Sedinger’s method. Angiography was performed with a 5F catheter to show the tumor staining and tumor-feeding arteries. Anticarcinogens including cisplatin (60 mg/m^2^) or oxaliplatin (60 mg/m^2^), 5-FU(600 mg/m^2^) or raltitrexed (4 mg) were infused into the arteries and 350–560 μm of gelatin sponge or polyvinyl alcohol were used to block all tumor-feeding arteries (**Figures 1D**, **2C, D**, **3C, D**).

### Follow Up

Patients underwent computed tomography examinations about 1, 3, and 6 months after the first TACE procedure (**Figures 1E, F**, **2E, F**, **3F**) and efficacy was evaluated according to the guidelines by Response Evaluation Criteria in Solid Tumors (RECIST) (10). Completely relieved was considered if all target lesions disappeared and partially relieved was considered if the tumor diameter decreased by 30% or more. Progressive development was defined as an increase in tumor diameter of 20% or greater. The objective response rate was defined as the sum of completely relieved and partially relieved. The disease control rate was defined as the sum of completely relieved, partially relieved, and stable development. All patients were followed up until death or loss to follow-up. Adverse reactions were evaluated using the National Cancer Institute Common Terminology Criteria for Adverse Events Version 3.0.

## Results

### TACE

Selective angiography showed that the tumor-feeding arteries appeared thickened and disordered, with irregular staining of the oral carcinoma, which vanished after embolization. During transcatheter perfusion, median dosages of cisplatin and oxaliplatin (n = 9) were of 90.0 mg and 100 mg, respectively; 5-FU or raltitrexed was 750 mg and 4 mg, respectively. Gelatin sponge (350–560 μm), polyvinyl alcohol (350–560 μm), and microspheres (300–500 μm) were used for embolization in eight, seven, and three patients, respectively. The microcoil (2 ∗ 20 mm) was used for one patient with massive oral hemorrhage. The median procedure time was 92.5 min (**Table 2**).

**Table 2:** Clinical data on TACE procedure and tumor diameter change.

Variables	TACE
Technique success	18 (100%)
Rebleeding	2 (11.1%)
Hospital stay, days	12.0 (10.0, 14.5)
Procedure time, min	92.5 (65.0, 120.0)
Medical cost, ×10^4^ ¥	3.4 (2.9, 4.7)
Complications	11 (61.1%)
Local pain	8 (44.4%)
Nausea or vomiting	3 (16.7%)
Fever	2 (11.1%)
Oral mucosal ulcers	1 (5.6%)
Tumor diameter, mm	
Before TACE	46.0 (30.5, 66.0)
1 month later	36.5 (22.8, 70.8)
3 months later	44.0 (23.0, 57.0)
6 months later	36.5 (15.0, 67.8)

¥, Renminbi (RMB) "yuan".

### Efficacy

A total of 31 sessions of TACE were performed on 18 patients, with a technical success rate of 100%. For 12 patients combined with oral hemorrhage, hemorrhage was effectively controlled in all patients after TACE, and two patients showed rebleeding 35 and 37 days later, with a clinical efficiency of hemostasis of 88.9%. Two patients received Iodine-125 implantation before or after TACE (**Figure 3E**). Six months after the first sessions of TACE, completely relieved, partially relieved, and stable development were observed in one, two, and three patients, respectively. The objective response rate and disease control rate were 20.0% and 86.7%, 38.5% and 61.5%, and 25.0% and 50.0% at 1, 3, and 6 months later, respectively (**Table 3**).

**Table 3:** Treatment responses of the study patients.

Response	1 month	3 months	6 months
Completely relieved	0 (0.0%)	0 (0.0%)	1 (8.3%)
Partially relieved	3 (20.0%)	5 (38.5%)	2 (16.7%)
Stable development	10 (66.7%)	3 (23.1%)	3 (25.0%)
Progressive development	2 (13.3%)	5 (38.5%)	6 (50.0%)
Objective response rate	3 (20.0%)	5 (38.5%)	3 (25.0%)
Disease control rate	13 (86.7%)	8 (61.5%)	6 (50.0%)

### Complication

All TACE procedures were safe, with no skin necrosis, cerebral infarction caused by ectopic embolization, or procedure-related death. Mild complications were observed in 11 patients (61.1%). Of these, mild-to-moderate local pain (44.4%) was the most common complication, which was easily relieved by treatment with analgesics. Nausea or vomiting and fever were observed in three and two cases, respectively.

### Follow Up

Seventeen patients (94.4%) were followed up, with a median duration of 37.8 months (IQR 22.3, 56.8). Nine patients died of tumor progression, one died of massive rebleeding, and one died of severe lung infection. The median overall survival was 23.8 months.

## Discussion

Half of the patients with oral carcinoma are diagnosed and managed as already being at a late stage, making the treatment challenging and difficult (11). Early-stage oral carcinomas can be effectively managed by surgery. However, tumor recurrence may be inevitable for some patients, and surgery has quite limited efficacy for patients with late-stage or recurrent oral carcinoma (12). Besides, traditional resection may damage oral function, and affect facial appearance, seriously affect the quality of life of patients. Conventional chemoradiotherapy is the main treatment strategy for patients with late-stage or recurrent oral carcinoma. However, medications in high doses may lead to severe adverse reactions and the clinical efficacy may be limited in cases of resistance. Due to the extensive involvement with or without metastatic lymph nodes, complete resection may be impossible for late-stage patients, with a high rate of recurrence after surgery (13).

Currently, TACE has been emerging as a palliative treatment for many kinds of late-stage carcinomas. By using local infusion of chemotherapeutic drugs at high dosage and embolization of tumor-feeding arteries for nutritional deprivation, TACE can cause tumor to shrink, necrosis, or even disappear (14–16). Besides, preoperative TACE may be beneficial to distinguish tumor boundaries from normal surrounding tissue and improve the resection rate by decreasing tumor volume and intraoperative bleeding (10), when compared with traditional treatments (17, 18).

Transcatheter infusion of high-dose anticarcinogens has been used for late-stage carcinoma of the head and neck, with or without concurrent radiation therapy (19–22). Regine et al. (18) reported that transcatheter cisplatin infusion is feasible for late-stage cancer of the head and neck if combined with hyperfractionated radiation therapy. Currently, there are few studies reporting the clinical outcomes of TACE for treating late-stage or recurrent oral carcinoma (6, 9). Kovaces et al. (9) reported that TACE using degradable starch microspheres and cisplatin showed a high overall response for late-stage cancer of the head and neck. Tomura et al. (6) performed TACE using carboplatin microcapsules for 14 patients with malignant tumors in the head and neck and achieved obvious tumor reduction.

In this study, the objective response rate and disease control rate were 20% and 86.7%, 38.5% and 61.5%, and 25% and 50% at 1, 3, and 6 months later, respectively. The median overall survival was 23.8 months. Besides, oral hemorrhage was effectively controlled in all patients after TACE. Theoretically, TACE allowed better efficacy by direct delivery of anticarcinogens into the tumor and lower adverse events by protecting the kidney, liver, and bone marrow from systemic effects (23). Mild complications were observed in this study, and local pain was the most common complication. This was similar to the study by Tomura et al. (6), in which only mild-to-moderate local pain was observed after TACE using carboplatin microcapsules.

There were some limitations to this study. This is a retrospective study with a long time study period of 2015–2021 and was only performed in a single center. The sample size of enrolled patients is small, and we could not avoid some bias for the evaluation of clinical outcomes.

### Conclusion

TACE is a safe and effective procedure with minimal invasiveness for the treatment of late-stage or recurrent oral carcinoma. TACE can be recommended as a palliative treatment, especially for patients with oral hemorrhage.

## Data availability statement

The original contributions presented in the study are included in the article/supplementary material. Further inquiries can be directed to the corresponding author.

## Ethics statement

The studies involving human participants were reviewed and approved by the Institutional Review Board of Zhengzhou University. The patients/participants provided their written informed consent to participate in this study.

## Author contributions

XH and JR conceptualized the study. YB, TD, and WP developed the methodology. XH, and JR validated the study. YB, TD, WP, FT, YW, and DJ performed the formal analysis. YB wrote and prepared the original draft. YB, TD, and WP wrote, reviewed, and edited the article. XH and JR conducted the visualization. XH and JR supervised the study. All authors listed have made a substantial, direct, and intellectual contribution to the work and approved it for publication.

## Conflict of interest

The authors declare that the research was conducted in the absence of any commercial or financial relationships that could be construed as a potential conflict of interest.

## Publisher’s note

All claims expressed in this article are solely those of the authors and do not necessarily represent those of their affiliated organizations, or those of the publisher, the editors and the reviewers. Any product that may be evaluated in this article, or claim that may be made by its manufacturer, is not guaranteed or endorsed by the publisher.
